# Smilagenin Protects Dopaminergic Neurons in Chronic MPTP/Probenecid—Lesioned Parkinson’s Disease Models

**DOI:** 10.3389/fncel.2019.00018

**Published:** 2019-02-05

**Authors:** Xuan He, Shuangshuang Yang, Rui Zhang, Lina Hou, Jianrong Xu, Yaer Hu, Rang Xu, Hao Wang, Yongfang Zhang

**Affiliations:** ^1^Department of Pharmacology, Institute of Medical Sciences, Shanghai JiaoTong University School of Medicine (SJTUSM), Shanghai, China; ^2^Scientific Research Center, Xinhua Hospital, Shanghai JiaoTong University School of Medicine (SJTUSM), Shanghai, China

**Keywords:** brain-derived neurotrophic factor, Chinese herb, dopaminergic neurons, glial cell line-derived neurotrophic factor, Parkinson’s disease

## Abstract

Current therapies for Parkinson’s disease (PD) only offer limited symptomatic alleviation but fail to hamper the progress of the disease. Thus, it is imperative to establish new approaches aiming at protecting or reversing neurodegeneration in PD. Recent work elucidates whether smilagenin (abbreviated SMI), a steroidal sapogenin from traditional Chinese medicinal herbs, can take neuroprotective effect on dopaminergic neurons in a chronic model of 1-methyl-4-phenyl-1,2,3,6-tetrahydropyridine (MPTP) conjuncted with probenecid mice. We reported for the first time that SMI significantly improved the locomotor ability of chronic MPTP/probenecid–lesioned mice. SMI increased the tyrosine hydroxylase (TH) positive and Nissl positive neuron number in the substantia nigra pars compacta (SNpc), augmented striatal DA and its metabolites concentration and elevated striatal dopamine transporter density (DAT). In addition, dopamine receptor D2R not D1R was down-regulated by MPTP/probenecid and slightly raised by SMI prevention. What’s more, we discovered that SMI markedly elevated striatal glial cell line-derived neurotrophic factor (GDNF) and brain-derived neurotrophic factor (BDNF) protein levels in SMI prevented mice. And we found that SMI increased GDNF and BDNF mRNA level by promoting CREB phosphorylation in 1-methyl-4-phenylpyridimium (MPP^+^) treated SH-SY5Y cells. The results illustrated that SMI could prevent the impairment of dopaminergic neurons in chronic MPTP/probenecid-induced mouse model.

## Introduction

Parkinson’s disease (PD) is an age-related debilitating neurodegenerative disorder, characterized pathologically by selective loss of dopaminergic neurons in the substantia nigra pars compacta (SNpc) accompanied by a decrease in striatal dopamine level, and intracytoplasmic Lewy bodies aggregated by phosphorylated α-synuclein (αSyn). Additionally, the clinical symptoms such as resting tremor, rigidity, slowness of initial movement do not fully present until there is a loss of 50%–60% SNpc neurons (Przedborski, 2017; Schapira et al., 2017). However current pharmacological therapies for PD, which dopamine replacement is a mainstay of therapeutic strategies, only alleviate symptoms but fail to hamper neurodegeneration process and restore dopaminergic dysfunction. What’s worse, the existing therapies for PD after long-term treatment could cause severe adverse effects, such as motor response fluctuations and dyskinetic movements (Tarazi et al., 2014; Pires et al., 2017; Wong and Krainc, 2017). Thus, it is greatly imperative to establish neuroprotective therapy available for PD.

Administration of neurotrophic factors such as brain-derived neurotrophic factor (BDNF) and glial cell line-derived neurotrophic factor (GDNF) are generally recognized as powerful survival factors for the degenerated dopaminergic neurons and the nigro-triatal pathway in PD (Cass et al., 2006; Kramer and Liss, 2015; Sun et al., 2015). Although the potential neuroprotective effects of these neurotrophic factors are undisputed, unfortunately in a randomized controlled trial of intraputamenal infusion of recombinant human GDNF in patients with PD, there is no remarkable improvement in patients receiving GDNF (Lang et al., 2006; Ibáñez and Andressoo, 2017). Owing to its large polypeptide structure and consequent poor bioavailability, it is difficult for these neurotrophic factors to permeate the blood brain barrier (Tarazi et al., 2014; Fan et al., 2016).

Consequently, it is greatly urgent to implement some strategies to stimulate the endogenous expression of neurotrophic factors after oral administration to slow down or reverse the progression of neuronal degeneration. Traditional Chinese medicine have accumulated much knowledge in treatment of PD so that it might be feasible to develop drugs from Chinese medicinal herbs for curing PD (Zhang et al., 2015). Smilagenin (Cogane; 5β, 2α, 25R-spirostan-3β-ol, abbreviated as SMI), with a molecular weight of 416.63 daltons, is a lipid-soluble small-molecule steroidal sapogenin from *Rhizoma anemarrhenae* and *Radix asparagi* widely used in traditional Chinese medicine for treating chronic neurodegeneration diseases (Visanji et al., 2008; Sy et al., 2016). Our previous studies have confirmed that SMI could not only protect the cultures of rat embryonic mesencephalic neurons from 1-methyl-4-phenyl-1,2,3,6-tetrahydropyridine (MPTP) toxicity *in vitro* but also enhance GDNF release as well as motor function of aged rat *in vivo* (Zhang et al., 2008; Li et al., 2013). However, whether SMI could protect dopaminergic neurons *in vivo* in chronic MPTP/probenecid-lesioned mice are unknown. To clarify the protecting effect of SMI on dopaminergic neuron we adopted the chronic MPTP/probenecid mouse model to investigate locomotor ability and the effects of SMI on nigrostriatal dopaminergic system as well as GDNF and BDNF expression (Petroske et al., 2001; Schildknecht et al., 2017; Nonnekes et al., 2018).

## Materials and Methods

### Materials

SMI with a purity of over 98 percent was supplied by Phytopharm plc. UK. One-methyl-4-phenyl-1,2,3,6-tetrahydropyridine hydrochloride (MPTP HCl), 1-methyl-4-phenylpyridimium (MPP^+^) was from Sigma, Dulbecco’s Modified Eagle Medium was from Gibco (Grand Island, NY, USA), probenecid, hydroxypropyl methyl cellulose (HPMC-Na), Ketanserin, SCH23390, fluoxetine and GBR-12909 and all reagents used in HPLC except acetonitrile were purchased from Sigma. Rabbit anti-mice tyrosine hydroxylase (TH) polyclonal antibody and 3,3′-diaminobenzidine (DAB) were from Chemicon. SABC kit was from Boster Bioengineering Co. Wuhan, China. [^125^I]-FP-CIT was synthesized using Na[^125^I] (from Chengdu Gaotong Isotope Co) and FP-CIT (from Jiangsu Institute of Nuclear Medicine) in our laboratory. [^3^H] SCH23390 (specific activity 80.5 Ci/mmol) and [^3^H] spiperone (specific activity 16.2 Ci/mmol) were purchased from Perkin Elmer Inc. Acetonitrile was from Merck. BDNF and GDNF ELISA kit were acquired from Promega company. Antibodies against the following proteins were used in the study: anti-BDNF (Abcam), anti-GDNF (Abcam), anti-DAT (Santa Cruz), anti-CREB (Santa Cruz), anti-pCREB (Santa Cruz), anti-D1 receptor (Millipore), anti-D2 receptor (Chemicon), anti-β-actin (Sigma). All primers used in qRT-PCR were designed using Primer Premier 5.0 software and synthesized by Shanghai Sangon Biotech Co. Ltd (Shanghai, China). The SYBR Green PCR Master Mix kit was from ABI (Warrington, UK).

### Production of Animal Models and Drug Administration

Male C57BL/6 mice (10 weeks old, 23.80 ± 1.32 g, from Shanghai SIPPR-BK Laboratory Animal Company) were housed five per cage and maintained on a 12 h light-dark cycle in standard conditions. The room temperature and relative humidity were set at 22 ± 2°C and 55% ± 15% respectively, with food and water available *ad libitum*. This study was carried out in accordance with the recommendations of the NIH Guide, Shanghai JiaoTong University Animal Ethic Committee. The protocol was approved by the Shanghai JiaoTong University Animal Ethic Committee. One week period of acclimatization was allowed between delivery of mice and commencement of treatment.

**Figure 1:**
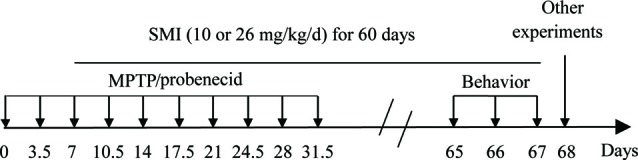
Time schedule of experiments *in vivo*.

The chronic PD model was produced according to the previous article with slight modifications (Petroske et al., 2001). In brief, 10 doses of MPTP HCL in saline plus probenecid in dimethyl sulfoxide were given in 5 weeks, with an interval of 3.5 days between consecutive injections. Each time, probenecid (250 mg/kg) was given by intraperitoneal injection 30 min prior to subcutaneous injection of MPTP (15 mg/kg; Figure 1). The normal control group was injected simultaneously with intraperitoneal injections of DMSO and 30 min later subcutaneous injections of saline. The mice were divided into four groups (seventeen in each group): normal control, MPTP/ probenecid (MPTP/P in figures) control, MPTP/ probenecid treated with SMI at dose of 10 and 26 mg/kg/day. From the third dose of MPTP injection, all mice were administered orally by intragastric administration either SMI or vehicle (HPMC) once daily for 60 days. For the last 3 days, locomotor ability of mice is evaluated by the rotarod and open field test.

### Rotarod and Open Field Test for Behavioral Assessment

We employed a modification of the procedure described by Rozas using a rotarod apparatus (IITC Life Science, Woodland Hills, CA, USA; Rozas et al., 1998). In general, all mice were trained to be accustomed to the rotarod apparatus at low speed rotation before formal experiment. Then each mouse was tested on rotating rods at a rate of six increments (8, 12, 16, 20, 24, 28 rpm) once per day for 3 days. Duration of each speed does not exceed 150 s. At least a 5 min resting period was required between each speed to alleviate mouse stress and fatigue. The overall rotarod performance for each mouse was obtained by plotting the average time on the rod at each speed vs. the rotating speed, and finally calculating the area under the curve (AUC) to evaluate the mice locomotor ability.

Next, we test spontaneous activity in a white open-field box. First the mice were put into the box to make it accustom to the environment for 5 min. Twenty-four hours later, the mice were placed into the same box and let them freely explore for 5 min. The locomotor activity was recorded by a video camera directly above the box and analyzed by a video track system (Noldus Ethovision).

### Tyrosine Hydroxylase Immunohistochemistry and Nissl Staining in the SNpc and Stereological Counting

Coronal sections through the substantia nigra were processed for TH immunohistochemistry and Nissl staining as described previously with minor modifications (Xu et al., 2010). Briefly, mice were euthanized and fixed by 4% paraformaldehyde. Coronal frozen sections (50 μm) from mid brain SN were treated with 0.3% hydrogen peroxidase in methanol and subsequently in 0.3% Triton X-100 and 5% bovine serum albumin. For TH immunostaining, the sections were then incubated with rabbit anti-mouse TH polyclonal antibody (1:600) at 4°C overnight. The following day, sections were treated with biotinylated anti-mouse IgG and then processed with avidin–biotin peroxidase complex. The peroxidase reaction was visualized by 0.05% DAB with 0.03% H_2_O_2_. For Nissl staining, sections were immersed in Nissl staining solution for 10 min. Finally, TH-positive and Nissl staining areas in SNpc were photographed with a Nikon TE300 inverted microscope. Meanwhile the total number of TH-positive neurons of TH-immunostained sections was quantified by unbiased stereological counting. Briefly, neurons were quantified using an Olympus BX61 microscope with a monitored x-y-z stage linked to the stereo investigator software in every fourth section of each mouse for a total of 8–9 slides through the entire SNpc. As described previously, the software could calculate total numbers of TH-positive neurons automatically (Petroske et al., 2001; Xu et al., 2010).

### HPLC Analysis of DA and Its Metabolites

The method adopted from Liang was made some modifications (Liang and Tang, 2006). In brief, striatum was rapidly dissected, weighed, homogenized and centrifuged. The final supernatant was stored at −70°C until HPLC assay. The concentrations of dopamine and its metabolites (DOPAC and HVA) were determined by a Waters 515 HPLC system (Milliford, MA, USA) with an electrochemical detector (HPLC-ECD) and a microbore ODS column (100 × 1 mm i.d.). The mobile phase consisted of 0.1 M NaH_2_PO_4_, 0.5 mM EDTA, 10 mM NaCl, 1 mM sodium octyl sulfate (pH 3.1) and 5% acetonitrile, and was pumped at 65 μl/min. The sample was diluted 1:4 with the mobile phase to neutralize the pH before chromatography. The calibration materials were assayed in parallel.

### Autoradiography of Striatal Dopamine Transporter

Striatal dopamine transporter density (DAT) binding were estimated by [^125^I]-FP-β-CIT binding autoradiography in coronal frozen sections (20 μm in thickness) prepared from right hemisphere including striatum region. Thawed sections were immersed in binding buffer (50 mM Tris, 120 mM NaCl, 5 mM KCl^−^) for 2 × 15 min at room temperature, then incubated in the same buffer containing 50 pM [^125^I]- FP-β-CIT and 100 nM fluoxetine (to inhibit serotonin binding to the [^125^I]- FP-β-CIT) for 2 h at 25°C to determine the total binding. Non-specific binding (NSB) was assessed in parallel slices with the addition of 100 μM GBR 12909 (dopamine uptake inhibitor). All sections that were thoroughly washed and air-dried were exposed to X-ray film (Kodak, USA) for 2 d at 4°C. Then the film was developed and fixed. Finally, quantitative analysis of the autoradiographs was performed with a Gel Documentation System (Bio-Rad) by circumscribing the area of striatum on the digitized image and measuring the mean gray level. DAT density was expressed as the mean value of optical density unit.

### Radioligand Binding Assay of Dopamine D1 and D2 Receptor Density in Striatum

Densities of Dl and D2 receptor were measured by the radioligand binding assay, using [^3^H] SCH23390 (SA: 80.5 Ci/mmol) for dopamine Dl receptor and [^3^H] spiperone (SA: 16.2 Ci/mmol) for dopamine D2 receptor binding, according to the method described previously with minor modifications (Popoli et al., 1998). In brief, the isolated striatum was homogenized in ice-cold buffer containing 50 mM Tris-HCl, 10 mM MgCl_2_, 0.25 M sucrose, and subsequently centrifuged at 2,000 *g* for 15 min, then at 27,000 *g* for 15 min at 4°C. The precipitate was suspended with the above buffer without sucrose, and mixed as a membrane protein suspension. Micro-Lowry’s method was utilized to quantify sample protein content. Dopamine receptor activity was measured in a parallel set of reaction tubes. A single dose of [^3^H] SCH23390 at a saturation concentration of 5 nM was selected based on preliminary multipoint saturation analysis for all samples to detect D1 receptor. Parallel tubes with additional 5 μM unlabeled SCH23390 were used for measurement of NSB. D2 receptors were measured using 1.5 nM [^3^H] spiperone combined with 50 nM ketanserin to block binding to serotonin receptors. NSB was determined in the presence of 80 nM haloperidol. The binding reaction system was incubated at 37°C for 50 min. The reaction was terminated by rinsing in ice-cold distilled water and harvested on a glass fiber filter which was then baked at 80°C, immersed in 0.6% b-PBD xylene scintillator and measured with a liquid scintillation counter (Beckman LS 6500). The density of receptors was determined using the following formula: Receptor density = (Total binding − NSB)/measure efficiency × 60 × specific × protein concentration).

### Measurement of BDNF and GDNF Content by ELISA

BDNF and GDNF content were determined using BDNF or GDNF Emax Immunoassay System according to the manufacturer’s guidelines. Briefly, the sample dissected from the striatum of unilateral hemisphere was sonicated in cold lysis buffer containing 137 mM NaCl, 20 mM Tris (pH 8.0), 0.5% TritonX-100, 10% glycerol and centrifuged at 10,000 *g* for 10 min at 4°C. Subsequently the supernatants were retained for assay. Then plates were coated with anti-BDNF or GDNF monoclonal antibody overnight. After nonspecific binding was blocked, each well added sample protein, BDNF or GDNF standards were incubated for 2 h (BDNF) or 6 h (GDNF) with shaking at room temperature. After washing, plates were incubated with polyclonal anti-human BDNF or anti-GDNF polyclonal antibody 2 h at room temperature or overnight at 4°C. Anti-IgY HRP-conjugated secondary antibody was added to the washed plates and incubated 1 h for BDNF and 2 h for GDNF at room temperature. Plates were then washed and incubated with TMB One Solution for 10 min or 15 min. Finally, plates were added 1 N hydrochloric acid to terminate the color change reaction and measured the absorbance at 450 nm using a plate reader. Data were expressed as picograms per milligram of protein.

### Western Blot

Striatum tissues were homogenized by ultrasonication in 10 volumes of ice-cold RIPA lysis buffer respectively. The homogenates were then centrifuged at 12,000 *g* for 5 min at 4°C. The supernatants were collected and total protein concentration was determined according to the Micro bicinchoninic acid (BCA) procedure. Protein samples (40 μg) were separated by electrophoresis on a 10% PAGE and transferred to PVDF membranes. The membranes were blocked with 5% nonfat dried milk for 2 h at room temperature and then incubated with primary antibody overnight at 4°C. The membranes were rinsed three times with Tris-Buffered Saline with Tween-20 (TBST) and then followed by HRP-conjugated anti-rabbit/mouse IgG for 2 h at room temperature. After rinsing with TBST, the immunocomplexes were visualized by enhanced chemiluminescence using the ECL kit according to the manufacturer’s instructions. Densitometric analyses were performed using ImageJ software.

#### Cell Culture and Treatment

The SH-SY5Y cell line was a kind gift from Dr. H. Zhang of the Shanghai Institute of Materia Medica who bought the cell line from ATCC (Manassas, VA, USA). The cells were seeded at 5 × 10^5^ cells/ml in bottles containing DMEM cultured at 37°C with 5% CO_2_. When the cells were 70–80% confluence, SMI was added at a final concentration of 10 μM. Twenty-four hours later, 1 mM MPP^+^ was added.

#### Transfection of SH-SY5Y Cells With CREB siRNA

CREB siRNA and control siRNA were designed as previous described. CREB siRNA: 5′-UACAGCUGGCUAACAAUGG-3′; control siRNA: 5′-UUCUCCGAACGUGUCACGU-3′; Control siRNA showed no significant match and thus served as a non-functional control. Transfection was carried out using the Fugene 6 Transfection Reagent kit (Roche) following instructions in the kit protocol. In brief, when the cells were 70% confluent, 80 nM siRNA and 1 μl transfection reagent were added to 10^5^ cells and were then incubated for 4 h to complete the transfection. GDNF and BDNF mRNA were examined 48 h later.

#### RT-PCR of GDNF and BDNF mRNA

In order to test the effects of MPP^+^, SMI and CREB on the expression of GDNF and BDNF, qRT-PCR was carried out using the SYBR Green dye method as described previously (Zhang et al., 2008) with the following primers:

- GDNF; F-5′-CGGGACTCTAAGATGAAGTTATGGGATGTCGTG-3′,
- R-5′-GGGTCAGATACATCCACACCGTTTAGCGGAATGC-3′,
- BDNF; F-5′-AGCTGAGCGTGTGTGACAGTATTAG-3′
- R-5′-ATTGCTTCAGTTGGCCTTTTGATAC-3′
- GAPDH; F-5′-GACCCCTTCATTGACCTCAACTACA-3′
- R-5′-TCTCGCTCCTGGAAGATGGTGATG-3′.

The amount of GDNF and BDNF mRNA in the SH-SY5Y cells were determined by real time qRT-PCR at 24 h after the addition of MPP^+^. Data were finally normalized by respective GAPDH values from the same run and expressed as percent of the control sample.

#### Statistical Analysis

All data were represented as mean ± SEM. Statistical comparisons of data among the different groups were performed by an analyses of variance (ANOVA) followed by a *post hoc* Newman–Keuls using the SAS software package. *P* < 0.05 was judged to have statistical significance.

## Results

### Effect of SMI on Rotarod and Open Field Test for Behavioral Ability in Chronic MPTP/Probenecid-Lesioned Mice

**Figure 2:**
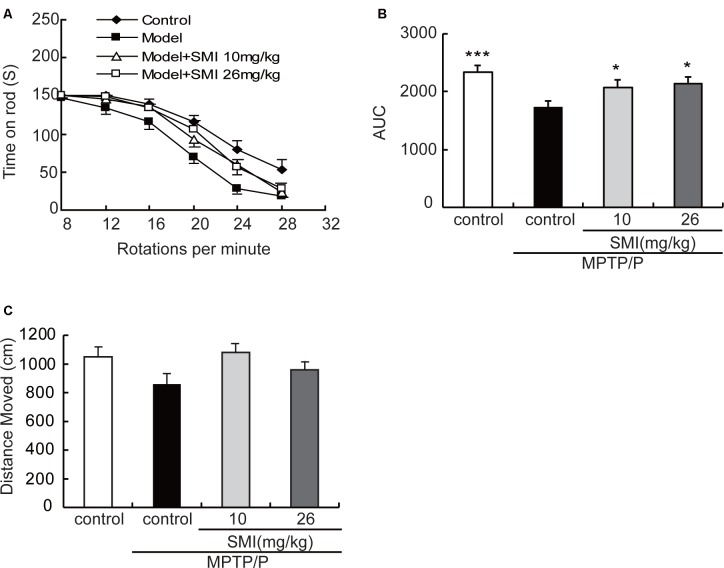
Effects of smilagenin (SMI) on behavioral test of chronic 1-methyl-4-phenyl-1,2,3,6-tetrahydropyridine (MPTP)/probenecid-lesioned mice. **(A)** Time on the rod vs. rotation speed, each point was the mean ± SEM of 3 days consecutive tests. **(B)** Statistical comparisons of area under the curve (AUC) values between groups. **(C)** Total distance moved, measured as centimeters moved in 5 min, data are expressed as mean ± SEM, *n* = 10. *P < 0.05 and ***P < 0.001 respectively as compared with untreated model group, respectively.

In the present study, we adopted a commonly utilized rotarod test to measure motor coordination in animals and the AUC to reflect the mice on the rotarod movement. The time stayed on the rods for all mice declined with the increase of rotational speed from 8 to 28 rpm in the rotarod apparatus. Generally, the curve dropped most rapidly in the MPTP/probenecid models, followed by SMI treatment, and the normal control mice declined slowest (Figure 2A). As indicated in Figure 2B, the AUC was significantly declined in MPTP/probenecid-injured mice when compared with normal controls (*P* < 0.001). Meanwhile, SMI at dose of 10 or 26 mg/kg/day significantly ameliorated locomotor ability of MPTP/probenecid-lesioned mice (*P* < 0.05).

As shown in Figure 2C, in the open field test, the total distance of MPTP/probenecid injured mice was less than that of control group, but there was no statistically significant difference. There was also no significantly improved locomotor activity with SMI treatment (*P* > 0.05).

### Effect of SMI on Tyrosine Hydroxylase Positive and Nissl Positive Neuron Number in Chronic MPTP/Probenecid-Lesioned Mice

**Figure 3:**
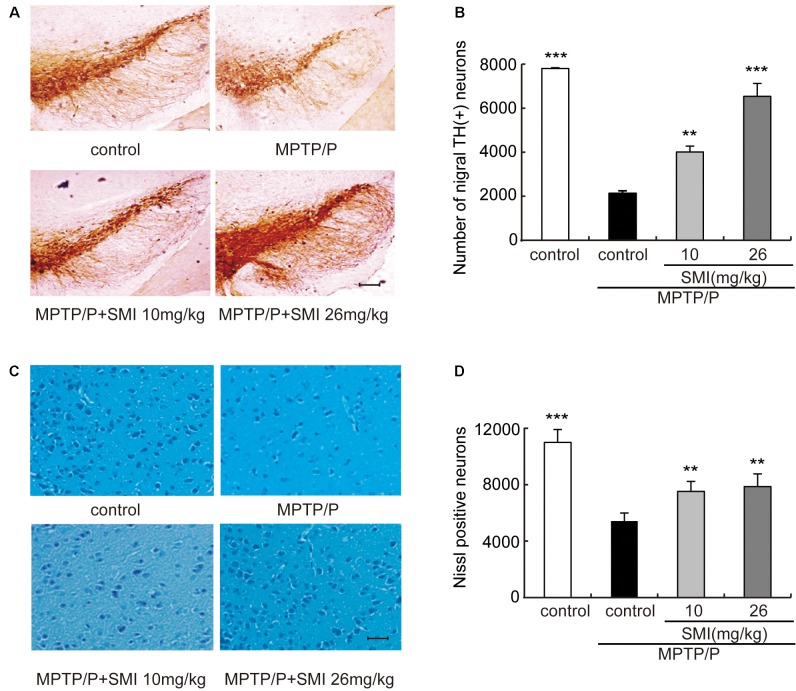
Effect of SMI on tyrosine hydroxylase (TH)-positive and Nissl positive neuron number in chronic MPTP/probenecid-lesioned mice. Panel **(A)** shows the representative slices of the control mice, model mice treated with vehicle, 10 mg/kg/day, and 26 mg/kg/day SMI successively. (×100, scale bar = 100 μm). Panel **(B)** shows the statistical results. Panel **(C)** shows the representative images of Nissl-stained neurons in the substantia nigra pars compacta (SNpc) of the control mice, model mice treated with vehicle, 10 mg/kg/d, and 26 mg/kg/day SMI successively (×100, scale bar = 100 μm). Panel (**D**) shows quantification of Nissl-stained neurons. Number of nigral TH-positive neuron and Nissl-stained neurons in each mouse was quantified at ×100 magnification and then normalized to its corresponding control. Data are expressed as mean ± SEM, *n* = 3. **P < 0.01 and ***P < 0.001, respectively as compared with untreated model group, respectively.

According to Figure 3A, we can see that the TH-positive neuron number of SNpc was decreased in the model mice compared with normal controls and was increased both in treatment with SMI at 10 and 26 mg/kg/day compared with model mice. Figure 3B has indicated the statistical results of TH-positive neuron numbers. Compared with normal control mice, TH-positive neuron number in the SNpc was reduced by 74.4% in chronic MPTP/probenecid injured model mice (*P* < 0.001). Two months’ treatment of 10 or 26 mg/kg/day SMI after three doses MPTP/probenecid injection increased TH (+) neurons by 104.1% and 228.8%, respectively when compared with MPTP/probenecid injection alone (*P* < 0.01, *P* < 0.001). On the other hand, Nissl staining showed that Nissl-stained neurons were reduced in the SNpc of chronic MPTP/probenecid injured model mice compared with controls and was increased after treatment with SMI in model mice (Figures 3C,D). These results suggest that administration of SMI may restore the loss of midbrain dopaminergic neurons caused by MPTP/probenecid.

### Effect of SMI on Striatal DA and Its Metabolites Concentration in Chronic MPTP/Probenecid-Lesioned Mice

**Figure 4:**
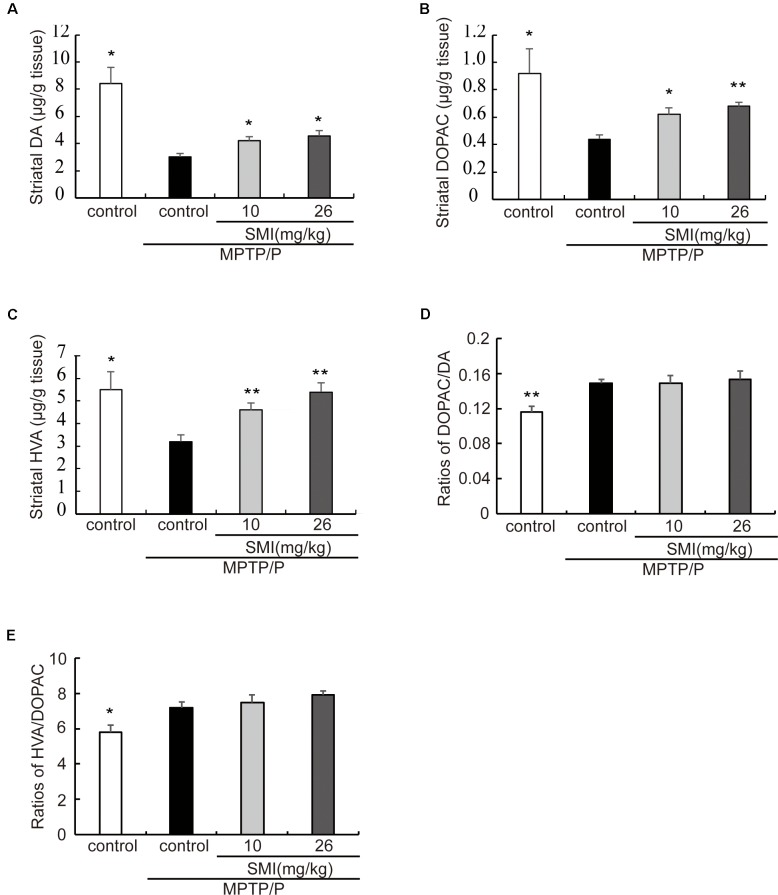
Effect of SMI on striatal DA and its metabolites concentration in chronic MPTP/probenecid-lesioned mice. Panels **(A–C)** show amount of DA and its metabolites in striatum detected by HPLC-ECD. Panel **(D)** shows ratios of DAPOC/DA. Panel **(E)** shows ratios of HVA/DOPAC. Data are expressed as mean ± SEM, *n* = 7. *P < 0.05 and **P < 0.01, respectively as compared with untreated model group, respectively.

In the chronic PD model induced by MPTP/probenecid, the average concentrations of DA in striatum was only 35.7% of controls (*P* < 0.05). After treatment PD model with SMI for 2 months at dose of 10 or 26 mg/kg/day, DA concentration increased to 50.1% and 54.5% of normal (*P* < 0.05; Figure 4A). Besides, the concentrations of the metabolites DOPAC and HVA, which were decreased in the MPTP/probenecid model, were also elevated by SMI (Figures 4B,C). For DOPAC/DA, the ratios were 0.149 ± 0.004, 0.149 ± 0.009 and 0.153 ± 0.010, respectively for model and model treated with SMI at dose of 10 or 26 mg/kg/day (Figure 4D). For HVA/DOPAC, the ratios were 7.19 ± 0.34, 7.50 ± 0.41 and 7.91 ± 0.23, respectively (*P* > 0.05; Figure 4E). We can see SMI has no significant effect on the ratios of DOPAC/DA and HVA/DOPAC. This implicates that SMI did not change the catabolism of DA.

### Effect of SMI on Striatal Dopamine Transporter Density and Protein Level in Chronic MPTP/Probenecid-Lesioned Mice

**Figure 5:**
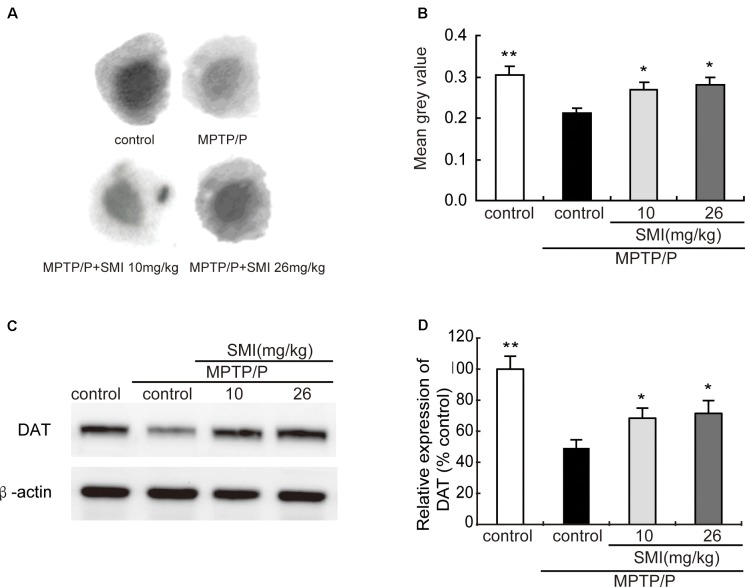
Effect of SMI on striatal dopamine transporter (DAT) density and protein level in chronic MPTP/probenecid-lesioned mice. Panel **(A)** shows representative autoradiographic images of the control mice, model mice treated with vehicle, 10 mg/kg/d, and 26 mg/kg/d SMI successively. Panel **(B)** shows the statistical results (*n* = 7). Panel **(C)** shows representative western blot bands of DAT. Panel **(D)** shows relative protein expression levels were quantified by densitometry analysis using Image J software on DAT bands (*n* = 4). Data are expressed as mean ± SEM. *P < 0.05 and **P < 0.01, respectively as compared with untreated model group, respectively.

Autoradiography has been utilized to clarify striatal DAT. Figure 5A was the representative autoradiographic images. The statistical results revealed in Figure 5B, striatal DAT density in MPTP/probenecid-treated mice was significantly reduced by 30.03% (*P* < 0.01), as compared with normal control mice. Additionally, after administration of SMI whatever 10 or 26 mg/kg/day in MPTP/probenecid-injured mice, DAT density in striatum increased by 26.42% and 32.55%, respectively when compared with MPTP/probenecid mice model alone (*P* < 0.05, *P* < 0.01). As shown in Figures 5C,D, the same trend was also observed through the Western blot analysis. We found that protein level of DAT significantly downregulated in MPTP/probenecid-treated mice as compared to controls (*P* < 0.01), which was elevated after treatment of SMI (*P* < 0.05).

### Effect of SMI on Striatal Dopamine D1 and D2 Receptor Density and Protein Level in Chronic MPTP/Probenecid-Lesioned Mice

**Figure 6:**
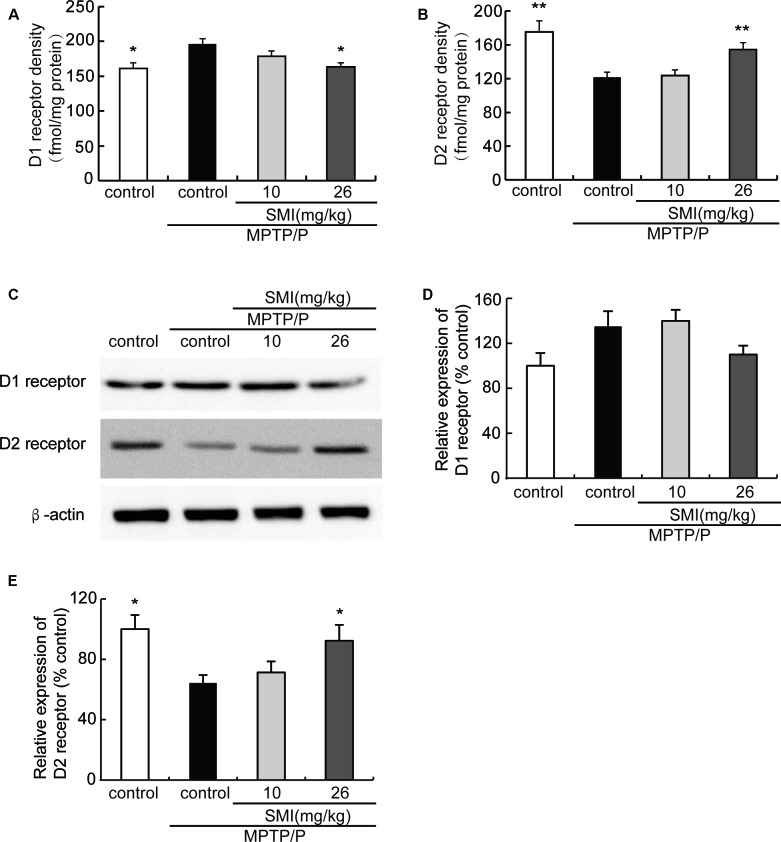
Effect of SMI on striatal dopamine D1 and D2 receptor density and protein level in chronic MPTP/probenecid-lesioned mice. **(A)** D1 receptor density, assessed by ^3^H-SCH23390 (*n* = 9). Panel **(B)** D2 receptor density, assessed by ^3^H-spiperone (*n* = 9). Panel **(C)** shows representative western blot bands of D1 and D2 receptor. Panels **(D,E)** show relative protein expression levels were quantified by densitometry analysis using Image J software on D1 and D2 receptor bands (*n* = 4). Data are expressed as mean ± SEM. *P < 0.05 and **P < 0.01, respectively as compared with untreated model group, respectively.

Saturation radioligand binding on striatal membranes using ^3^H-SCH23390 displayed increased D1 receptor density in the model mice compared with normal control and treatment with SMI at dose of 26 mg/kg/day (*P* < 0.05). SMI treatment produced D1 receptor downregulation from 196 to 164 fmol/mg protein in model mice (Figure 6A). Conversely, D2 receptor number in MPTP/probenecid-lesioned mice decreased compared with normal (*P* < 0.05); while after administration of SMI at dose of 26 mg/kg/day, there was a significantly increase (*P* < 0.01; Figure 6B). As shown in Figures 6C,D, we found that protein level of D1 receptor had no significant change through western blot and the reason might be antibody detection was not sensitive enough (*P* > 0.05). On the other hand, D2 receptor expression was downregulated in MPTP/probenecid-treated mice as compared to controls. Meanwhile, SMI at dose of 26 mg/kg/day, not 10 mg/kg/day, significantly restored D2 receptors protein level of MPTP/probenecid-lesioned mice (*P* < 0.05; Figure 6E).

### Effect of SMI on Striatal GDNF and BDNF in Chronic MPTP/Probenecid-Lesioned Mice

**Figure 7:**
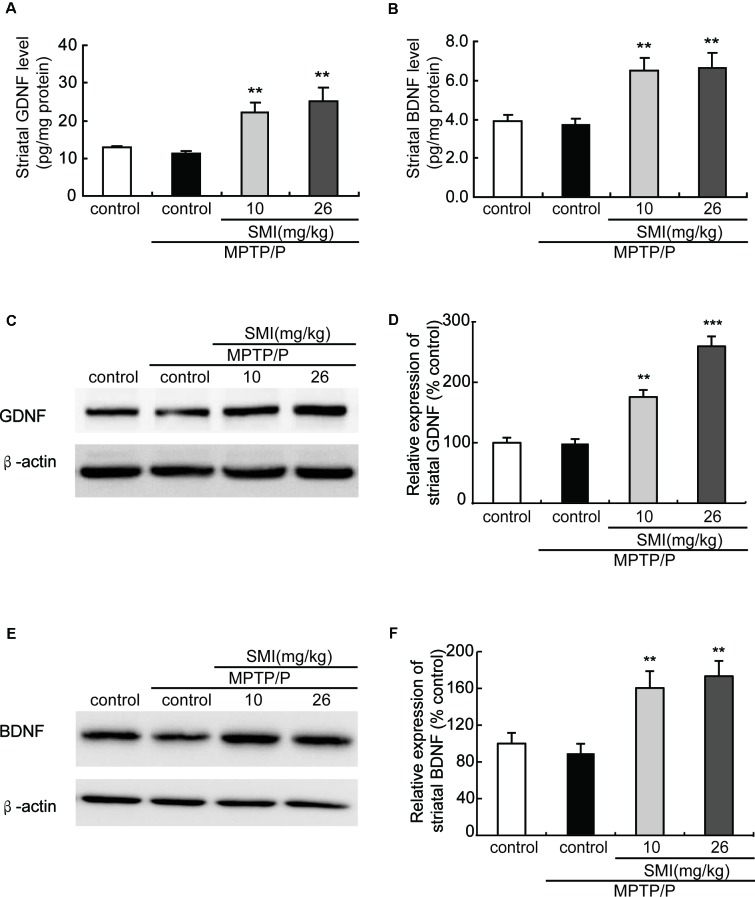
Effect of SMI on striatal glial cell line-derived neurotrophic factor (GDNF) and brain-derived neurotrophic factor (BDNF) levels in chronic MPTP/probenecid-lesioned mice. **(A)** Striatal GDNF levels by ELISA. **(B)** Striatal BDNF levels by ELISA. Panels **(C,E)** show representative western blot bands of GDNF and BDNF. Panels **(D,F)** show relative protein expression levels were quantified by densitometry analysis using Image J software on GDNF and BDNF bands (*n* = 4). Statistical comparisons between groups, data are expressed as mean ± SEM, **P < 0.01 and ***P < 0.001, respectively as compared with untreated model group, respectively.

Additionally, to further clarify the underlying mechanism of SMI’s neuroprotective effect, we applied ELISA and Western blot to detect striatal GDNF and BDNF protein content. We found that there was a slight reduction but no significant difference in protein contents of GDNF and BDNF in the striatum between the MPTP/probenecid-treated mice and normal control mice (*P* > 0.05; Figures 7A–F). As shown in Figures 7A,B, SMI administration elevated the striatal GDNF and BDNF protein content of MPTP/probenecid-induced mice by ELISA analysis (*P* < 0.01). The same trend was also observed through the Western blot analysis. Meanwhile, we found SMI at dose of 26 mg/kg/day more significantly increased the protein level of GDNF compared with lower dose (*P* < 0.001; Figure 7D).

### Effect of SMI on CREB Phosphorylation in Chronic MPTP/Probenecid-Lesioned Mice and CREB siRNA on mRNA Expression of GDNF and BDNF in MPP^+^ Treated SH-SY5Y Cells

**Figure 8:**
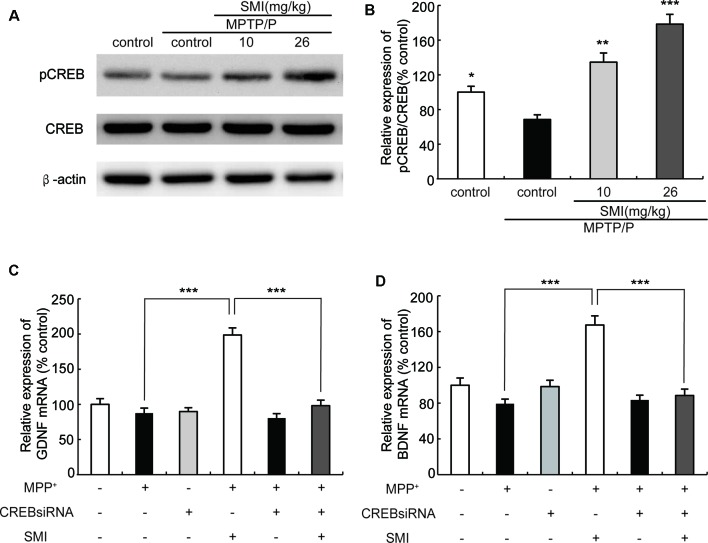
Effect of SMI on cyclic AMP responsive element binding (CREB) phosphorylation in chronic MPTP/probenecid-lesioned mice and CREB siRNA on mRNA expression of GDNF and BDNF in MPP^+^ treated SH-SY5Y cells. Panel **(A)** shows western blot bands of CREB and phosphorylation ofCREB (pCREB). Panel **(B)** shows relative expression of pCREB/CREB were quantified by densitometry analysis using Image J software on CREB and pCREB bands (*n* = 4). Data are expressed as mean ± SEM, *P < 0.05, **P < 0.01 and ***P < 0.001, respectively as compared with untreated model group, respectively. Panels **(C,D)** show relative expression of GDNF and BDNF mRNA by real time PCR. Data are expressed as mean ± SEM, ***P < 0.001.

Cyclic AMP responsive element binding protein (CREB) is a transcription factor involved in regulation of genes associated with synaptic and neural plasticity. The phosphorylation of CREB (pCREB) was necessary for active transcription. CREB has been reported to up-regulate the expression of a set of genes including BDNF and GDNF (Cen et al., 2006; Wang et al., 2017). As shown in Figure 8A, Western blot revealed that the expression of pCREB/CREB was reduced in MPTP/probenecid-treated mice as compared with normal control mice (*P* < 0.05). We found SMI treatment elevated the level of CREB phosphorylation and the differences between SMI at dose of 26 mg/kg/day and MPTP control was highly significant (*P* < 0.001; Figure 8B). We speculated SMI up-regulated the expression of GDNF and BDNF by increasing the level of CREB phosphorylation. To test this possibility, we blocked CREB by CREB siRNA in MPP^+^ treated SH-SY5Y cells and detected mRNA expression of GDNF and BDNF. We found that there was no significant difference in mRNA expression level of GDNF and BDNF between MPP^+^ treated cells and normal control. However, SMI treatment significantly increased GDNF and BDNF transcription in MPP^+^ treated cells (*P* < 0.001). After application of CREB siRNA, the elevation effect of SMI on mRNA expression of GDNF and BDNF was abolished. There was no significantly upregulation after SMI treatment (*P* > 0.05, Figures 8C,D).

## Discussion

It is for the first time that we have evaluated the neuroprotective effects of SMI as well as its effect on locomotor ability in chronic MPTP conjuncted with probenecid lesioned mice model of PD. Given SMI requiring long-term administration to exert well protective effects, the chronic MPTP/probenecid mice model which closely mimics the chronic and progressive neurodegeneration and behavioral deficits observed in human PD was more appropriate for investigating the drug’s potent neuroprotective strategies (Peoples et al., 2012; Schildknecht et al., 2017; Nonnekes et al., 2018). Following 60 days’ oral administration of SMI when the third dose of MPTP/probenecid injection afterwards.

It was demonstrated that MPTP/probenecid-intoxicated mice gave rise to impaired rotarod performance assessed by means of the rotarod test which is one of the most commonly utilized test to measure motor coordination in animals (Rozas et al., 1998; Petroske et al., 2001; Ayton et al., 2013). In the present study, we observed that rotarod performance was impaired in MPTP/probenecid-lesioned mice compared with the normal control and SMI administration improved MPTP/probenecid-induced motor deficits. The locomotor disorder is considered to be related to the injure of nigrostriatal dopaminergic system.

TH, DA, DAT and dopamine receptors are important hallmarks of the neurodegenerative alterations of nigro striatal dopaminergic neuron in PD (Peoples et al., 2012; Naskar et al., 2015; Zhang et al., 2018). We observed in this article that TH-positive and Nissl positive neuron numbers in SNpc markedly reduced in MPTP-lesioned mice consistent with previous research reported (Alam et al., 2017; Peng et al., 2018), which can be elevated by administration of SMI starting at the early stage of the model production. TH is a marker of dopaminergic neurons and Nissl body is a marker of neurons. SMI increases the number of TH-positive neuron and Nissl staining cells in the substantia nigra of model mice, reflecting the increase in the number of dopaminergic neurons in the substantia nigra. In addition, the amount of TH in the substantia nigra is increased, which is beneficial to the synthesis of DA. Furthermore, SMI treatment increases striatal DA level of chronic PD mice model and does not change its metabolites concentration. We could speculate that SMI may protect striatal DA at a relatively constant level and does not affect the rate of DA renewal by protecting residual neurons (Xu et al., 2010; Sun et al., 2012). In the case of chronic PD mice model, striatal DAT density was lowered when compared with the vehicle-treated mice (Anandhan et al., 2012; Hong et al., 2015; Miville-Godbout et al., 2016). SMI treatment can reverse the decline of DAT density. This facilitates the ingestion of DA to prepare next release after its release into the synaptic cleft and exerting physiological effects. In addition, DAT is specifically expressed in dopaminergic neurons. Due to the protection of dopamine neurons by SMI, DA synthesis was not significantly reduced, thus avoiding the D1 receptor hypersensitivity and increase in number to some extent. In addition, the up-regulation of D2 receptor density by SMI suggests that SMI may be used in combination with levodopa to improve the down-regulation of D2 receptor density induced by the long-term use of levodopa (Morissette et al., 1996). Thus, we could speculate that SMI exerts effects of elevating nigral TH positive neurons and striatal DAT density in chronic MPTP-intoxicated mice, suggesting that it possesses action of improving dopaminergic neuron loss induced by MPTP and preventing or slowing the process of neuronal degeneration. We also found there was a recovery phenomenon appeared in MPTP/probenecid treated mice after 6 months which suggested us that we could observe the role of SMI in MPTP/probenecid treated mice for a longer period of time and get more information (Petroske et al., 2001).

Although there is undisputed about its significance of the potential neuroprotective effects of both BDNF and GDNF in preclinical studies and GDNF has been shown in some PD patients to have a positive effect (Gill et al., 2003; Love et al., 2005; Lu et al., 2013), unfortunately owing to large polypeptide structure and consequent poor oral bioavailability of exogenous BDNF, in clinical studies using BDNF as a therapeutic agent have been inconclusive (Thoenen and Sendtner, 2002). It is greatly urgent to execute potential strategy to stimulate the endogenous expression of neurotrophic factors after oral administration to slow down or reverse the progression of neuronal degeneration. Our previous study *in vitro* indicated that SMI relied on stimulating the intrinsic GDNF expression to exert neuroprotective effect on primary cultured mesencephalic dopaminergic neurons (Zhang et al., 2008). Hence, we evaluated whether SMI could have an impact on chronic MPTP/probenecid-induced mice *via* enhancing expression of BDNF and GDNF in striatum. In this article, we observed that there were no alterations in striatal BDNF and GDNF protein levels between chronic MPTP/probenecid lesioned mice alone and the vehicle treated mice. However, long-term administration of SMI greatly elevated GDNF and BDNF content in the striatum of MPTP/probenecid-lesioned mice. In addition, we used MPP^+^-treated SH-SY5Y cells as an *in vitro* PD model to investigate the mechanism of SMI effect on GDNF and BDNF. We observed SMI treatment significantly increased GDNF and BDNF transcription in MPP^+^ treated cells. We also found CREB siRNA abolished the elevation effect of SMI on mRNA expression of GDNF and BDNF in MPP^+^-treated cells. We could speculate that SMI increased GDNF and BDNF protein level through promoting the phosphorylation of upstream transcription factor CREB and to increase mRNA expression of GDNF and BDNF. Presently there is a body of evidence revealing that BDNF and GDNF has strong neuroprotective effects on nigrostriatal dopaminergic neurons of animal PD models with 6-OHDA or MPTP, led to a substantial increase in the number of TH-positive neurons in SNpc as well as ameliorated behavioral disorders, stimulated TH positive fiber sprouting and elevated DA content (Biju et al., 2010; Ren et al., 2013). In the present research, it is reasonable to assume SMI protect and repair nigrostriatal dopaminergic system, at least in part, by promoting the phosphorylation of CREB to increase expression of endogenous GDNF and BDNF. GDNF and BDNF exert their neuroprotective effect to increase nigral TH positive neurons and striatal DAT density and subsequently ameliorate motor deficits in chronic MPTP/probenecid-intoxicated mice.

## Conclusion

In summary, this study demonstrates that administration of the SMI to chronic MPTP/probenecid animals improved the pathological development of disease. The possible mechanism that SMI exerted neuroprotective effects may be partially related to up-regulating CREB phosphorylation which triggers endogenous neurotrophic factors, and ameliorating nigrostriatal dopaminergic system, which is likely to halt the ongoing progression.

## Data Availability

All datasets generated for this study are included in the manuscript.

## Author Contributions

YZ, HW and RX conceived the study. XH and SY performed the experiments and data analyses. RZ, LH and JX provided intellectual inputs. XH and SY wrote the manuscript. All authors edited and approved the manuscript.

## Conflict of Interest Statement

The authors declare that the research was conducted in the absence of any commercial or financial relationships that could be construed as a potential conflict of interest.

## Abbreviations

SMI: smilagenin
MPTP: 1-methyl-4-phenyl-1,2,3 6-tetrahydropyridine
DAT: dopamine transporter density
GDNF: glial cell line-derive neurotrophic factor
BDNF: brain-derived neurotrophic factor
SNpc: substantia nigra pars compacta
α-syn: α-synuclein
PD: Parkinson’s disease
TH: tyrosine hydroxylase.
